# Comparison of paired human nasal and bronchial airway epithelial cell responses to rhinovirus infection and IL-13 treatment

**DOI:** 10.1186/s40169-018-0189-2

**Published:** 2018-05-02

**Authors:** Nicole Roberts, Reem Al Mubarak, David Francisco, Monica Kraft, Hong Wei Chu

**Affiliations:** 1 Department of Medicine, National Jewish Health, 1400 Jackson Street, Room A639, Denver, CO 80206 USA; 2 Department of Medicine, University of Arizona, Tucson, AZ USA

**Keywords:** Epithelial cells, Rhinovirus, IL-13, IP-10, Eotaxin

## Abstract

**Background:**

Because of its advantage as a minimally invasive procedure, nasal brushings have been increasingly used and proposed as a valuable approach to study lower airway diseases in lieu of bronchial epithelial cells. However, there is limited or conflicting evidence pertaining to whether nasal samples can be surrogates to bronchial samples. The goal of the present study is to test whether nasal epithelial cells have similar antiviral and inflammatory responses to IL-13 treatment and rhinovirus infection, a condition mimicking virally induced asthma exacerbation. Nasal and bronchial airway epithelial cells taken from the same patient were cultured under submerged and air–liquid interface (ALI) culture in the absence or presence of rhinovirus and IL-13 treatment. Inflammatory cytokines IP-10 and eotaxin-3, antiviral gene Mx1 and viral levels were measured.

**Results:**

In the absence of IL-13 treatment, nasal and bronchial cells showed a similar IP-10 response in both ALI and submerged cultures. Under the ALI culture, short term (e.g., 3 days) IL-13 treatment had a minimal effect on viral and Mx1 levels in both cell types. However, prolonged (e.g., 14 days) IL-13 treatments in both cell types decreased viral load and Mx1 expression. Under the submerged culture, IL-13 treatment in both cell types has minimal effects on viral load, IP-10 and Mx1. IL-13-induced eotaxin-3 production was similar in both types of cells under either submerged or ALI culture, which was not affected by viral infection.

**Conclusions:**

Our data suggest that nasal epithelial cells could serve as a surrogate to bronchial epithelial cells in future studies aimed at defining the role of type 2 cytokine IL-13 in regulating pro-inflammatory and antiviral responses.

## Background

A bronchoscopy is an adequate technique for physicians and scientists to obtain airway cells (e.g., bronchial brushings) or tissues from a patient’s lower airway to diagnose diseases and perform basic and translational research. However, there are risks following a bronchoscopy including bronchial spasms, bleeding and infection [1]. A minimally invasive approach such as nasal brushings is urgently needed to study lower airway diseases such as asthma. Though previous efforts have been taken to determine the surrogacy of nasal epithelial cells, there is no solid conclusion as to whether they can be ideal surrogates for bronchial epithelial cells.

The criteria for nasal cells to be surrogates for bronchial cells depend on the research questions being asked. In asthma research, many studies focus on the mechanisms of acute exacerbations associated with human rhinovirus (HRV) infection [2]. A common in vitro model to mimic asthmatic airway infection is to expose IL-13-treated airway (bronchial or nasal) epithelium to HRV (e.g., HRV16). So far, few studies have utilized paired bronchial and nasal cells to address the suitability of nasal cells as a surrogate of bronchial cells given the impact of the genetic factors on cellular responses. Moreover, due to the differences in mucociliary differentiation of airway cells grown under submerged versus air–liquid interface (ALI) culture, research findings using either method in different publications may not be comparable. Thus, both culture methods should be used in the same study for a better comparison of these two cell types. For example, Lacgowicz-Scroggins et al. [3] performed a study with human tracheobronchial epithelial cells in ALI and stimulated with IL-13 and HRV16. They found that differentiated cells had an increase in viral load when treated with IL-13, the virus was more likely to infect goblet cells over ciliated cells. In contrast, when the same experiment was done in submerged culture, they found no increase in viral load in the presence of IL-13. This effect has not been confirmed in paired nasal epithelial cells. Conflicting results have been reported as to the interchangeability of these two cell types. Lopez-Souza et al. [4] showed that bronchial epithelial cells were more susceptible to HRV infection than nasal epithelial cells, which was associated with greater induction of ICAM-1 (a HRV16 receptor) following viral infection in bronchial epithelial cells. McDougall et al. [5] concluded that nasal brushings could be surrogates for bronchial brushings based on the production of various mediators such as IL-8, IL-6 and morphology following stimulation with pro-inflammatory cytokines such as IL-1β and TNF-α. Thavagnanam et al. have done similar experiments with nasal and bronchial pairs in asthmatic child samples using IL-13 as a stimulus and found similar cytokine responses of both cell types to IL-13 [6]. However, HRV infection was not performed in this publication.

In the current study, we aimed to use paired nasal and bronchial cells cultured in both submerged and ALI conditions to determine whether cells from nasal brushings can be representative of those from bronchial brushings in the study of airway responses to HRV infection in a type 2 cytokine milieu. We focused on several outcomes that are highly relevant to acute exacerbations associated with HRV infection in asthma patients. These include interferon gamma-induced protein 10 (IP-10), a pro-inflammatory cytokine associated with active viral infections in asthma patients [7], eotaxin 3, a chemokine for eosinophilic inflammation, viral load and an antiviral gene Mx1, one of the interferon-stimulated genes. Our findings are supportive of our hypothesis that nasal and bronchial epithelial cells have similar responses to HRV16 and IL-13 treatments.

## Methods

### Materials

NIH 3T3 fibroblasts were from The American Type Culture Collection (ATCC). Transwells were purchased from Corning Incorporated, Coring, NY. Bovine collagen I was obtained from Advanced BioMatrix (Carlsbad, CA). Submerged cell culture media consisted of Bronchial Epithelial Cell Growth Medium (BEGM) with antibiotics from Lonza (Walkersville, MD). Air–liquid interface (ALI) media consisted of the PneumaCult kit purchased from Stem Cell Technologies (Vancouver Canada). Recombinant human IL-13 from R&D systems, Minneapolis, MN was reconstituted in 0.1% bovine serum albumin (BSA) aliquots and stored at − 80 °C. Human rhinovirus 16 (HRV16) was propagated in H1-Hela cells (ATCC) and purified as previously described [8]. RNA lysis buffer (RLT) was from Qiagen (Hilden, Germany). IL-8 and IP-10 ELISA kits were purchased from R&D systems (Minneapolis, MN).

### Isolation and culture of human bronchial and nasal epithelial cells

Paired nasal and bronchial brushings were performed in human subjects who needed the bronchoscopy for diagnosis purposes (Table 1). Subjects presenting with differing disease states were used in order to broaden the implications of our findings for comparing nasal and bronchial epithelial cells. They were consented through the National Jewish Health (NJH) Biobank honest broker system. Collection of the samples was approved by the Institutional Review Board at NJH (IRB # HS 3114) and the University of Arizona College of Medicine. Bronchial brushings were collected during the bronchoscopy and saved in PBS for cell isolation [9]. After the bronchoscopy, nasal brushings were collected from the inferior turbinate and saved in PBS for cell isolation.

**Table 1:** Subjects used in study

ID	Gender	Age (Years)	Smoking status	Diagnosis	FEV1 (%)
1	Female	45	Past	Acute sarcoidosis	92
2	Female	61	Never	Chronic cough	77
3	Male	27	Never	Healthy	99
4	Female	67	Never	Intermittent bronchiectasis	65
5	Female	50	Past	Osteoporosis	65

Nasal and bronchial epithelial cells were initially expanded onto irradiated 3T3 fibroblasts (passage 0) and then further passed to grow into passage 1 (P1) which was later frozen in liquid nitrogen. Nasal and bronchial epithelial cells from P1 vials were expanded into P2 cells onto irradiated 3T3 fibroblasts in the presence of a Rho kinase inhibitor Y-27632 as described by us and others [10, 11]. These P2 cells were then put onto collagen coated transwells in 12 well plates for ALI culture, and in collagen-coated 12 well plates for submerged culture.

**Fig. 1:**
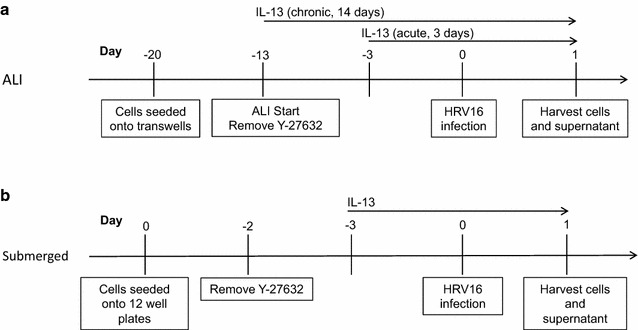
Timeline for ALI cultures (**a**) and submerged cultures (**b**) of paired human primary bronchial and nasal epithelial cells

**Fig. 2:**
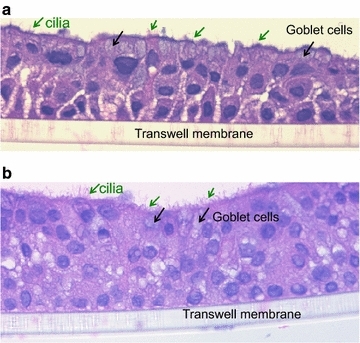
Mucociliary differentiation of brushed bronchial (**a**) and nasal (**b**) epithelial cells grown at the air–liquid-interface for 14 days. H&E staining, original magnification × 400

For the ALI culture, cells on transwells were given 7 days to proliferate and become 100% confluent [12]. Cells were treated with IL-13 for 14 days (chronic) starting on the first day of ALI, or for 3 days (acute) starting on day 11 of ALI (Fig. 1 a, b). The rationale for starting the chronic IL-13 (14 day) treatment at the beginning of ALI was to reflect the nature of injured airways of asthmatics. Damage of airway epithelium (e. destruction of ciliated cells) has been reported in severe asthma subjects [13, 14]. As a result, the basal cells will be exposed. Treating un-differentiated cells with IL-13 on day 0 of ALI was intended to reflect the damaged epithelium. On the viral infection day, cells were washed six times with Dulbecco’s PBS (DPBS) and then infected with HRV16 (10^6^ PFU/transwell) for 40 min at room temperature in a sterile hood and then 90 min at 34.5 °C. After infection, each well was washed eight times with DPBS and then given fresh media to sit overnight at 37 °C. After 24 h of viral infection, basolateral supernatants were saved for cytokine analysis and cells were saved in RLT for RNA extraction. Figure 2 demonstrates the mucociliary differentiation of bronchial and nasal epithelial cells at the ALI culture.

Submerged cultures of nasal and bronchial epithelial cells were carried out for 48 h to yield approximately 80% confluent. Cells were then treated with IL-13 (10 ng/ml) 48 h before infection with HRV16 (10^5^ PFU/well) in culture media (BEGM). The cells were washed once with PBS before infection and then left for 2 h in a 37°C incubator with 5% CO_2_ for 2 h. After infection, the cells were washed twice with PBS and fresh media was added back onto the cells. After 24 h of infection, supernatants were collected and saved for cytokine analysis while cells were saved in RLT for RNA extraction. Chronic IL-13 treatment (e.g., 14 days of treatments) was not done in submerged culture because they would undergo the aging process, which could compromise our research conclusions.

### ELISA for human Eotaxin 3 and IP-10

Eotaxin 3 and IP-10 levels were measured in supernatants of cultured epithelial cells in the ALI as well as the submerged conditions using specific DuoSet ELISA kits (R&D Systems) as per manufacturer’s instructions.

### Quantitative real-time RT-PCR

Real-time PCR was performed on the CFX96 (Bio Rad) using TaqMan gene expression assays made by Applied Biosystems (Life Technologies, Foster City, CA). Threshold cycle (Ct) levels were identified for HRV and Mx1, as well as housekeeping gene 18S rRNA (ALI culture) and GAPDH (submerged culture). Relative mRNA expression was calculated using delta Ct method and normalized to the housekeeping gene.

### Statistical analysis

Paired *t* test was used to compare treated versus non-treated conditions in nasal and bronchial cells using the Prism GraphPad software. A p value < 0.05 was considered significant.

## Results

### Effects of IL-13 treatment on IP-10 expression and viral load in bronchial epithelial cells

**Fig. 3:**
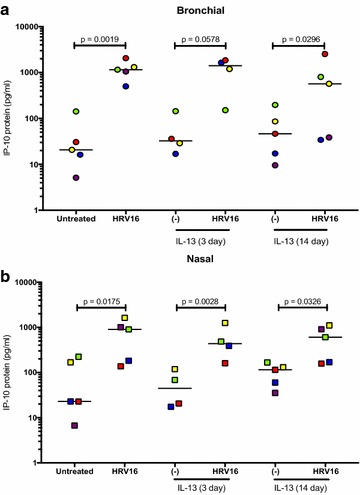
Human rhinovirus 16 (HRV16) infection increases IP-10 in paired human primary bronchial (**a**) and nasal (**b**) epithelial cells cultured at air–liquid interface (ALI). Each of the colored solid circles (n = 4–5 subjects) represents the data from an individual subject. The horizontal lines indicate medians

**Fig. 4:**
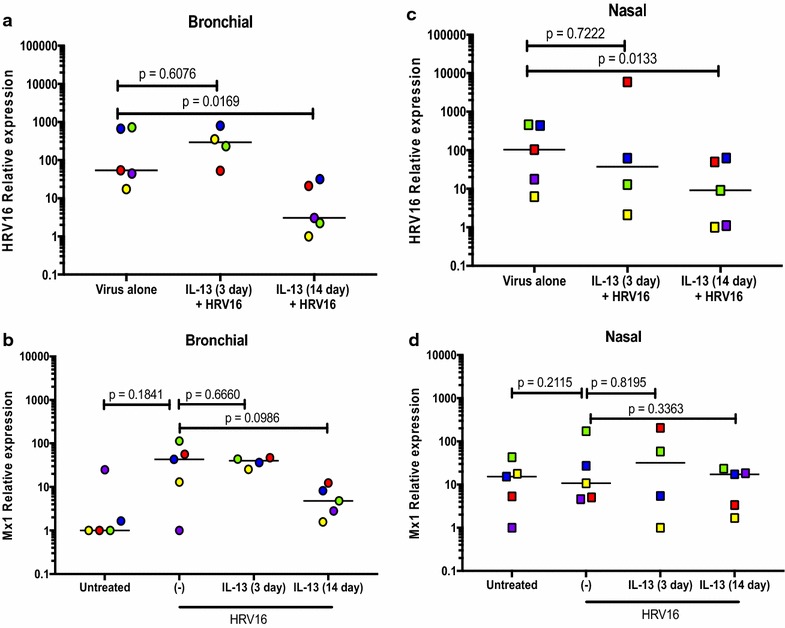
Effects of IL-13 treatment on human rhinovirus 16 (HRV16) infection in air–liquid interface (ALI) cultures of paired human primary bronchial and nasal epithelial cells. Viral load and Mx1 mRNA expression were measured in bronchial (**a**, **b**) and nasal (**c**, **d**) epithelial cells exposed to acute and chronic IL-13 treatments and/or HRV16. Each of the colored solid circles (n = 4–5 subjects) represents the data from an individual subject. The horizontal lines indicate medians

In the ALI culture, IP-10 significantly increased after HRV16 infection (Fig. 3a). IL-13 treatment alone did not affect IP-10 production. Treatment with IL-13 for 3 days (acute IL-13 model) or for 14 days (chronic IL-13 model) did not significantly change IP-10 induction by the viruses. Moreover, there were no significant effects of acute vs. chronic IL-13 treatment on IP-10 production following HRV16 infection. The viral load as detected by PCR was reduced by the chronic (p < 0.05), but not the acute IL-13 treatment in ALI cells (Fig. 4a). Antiviral gene Mx1 expression followed the similar trend of viral load, but was not significantly different (Fig. 4b).

**Fig. 5:**
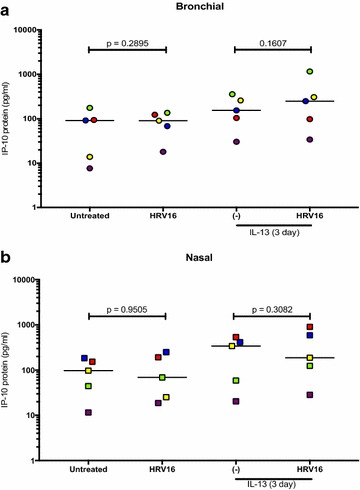
IP-10 production in paired human primary bronchial (**a**) and nasal (**b**) epithelial cells under the submerged culture. IP-10 was measured in supernatants of cells treated with or without IL-13 and/or human rhinovirus (HRV16). Each of the colored solid circles (n = 5 subjects) represents the data from an individual subject. The horizontal lines indicate medians

**Fig. 6:**
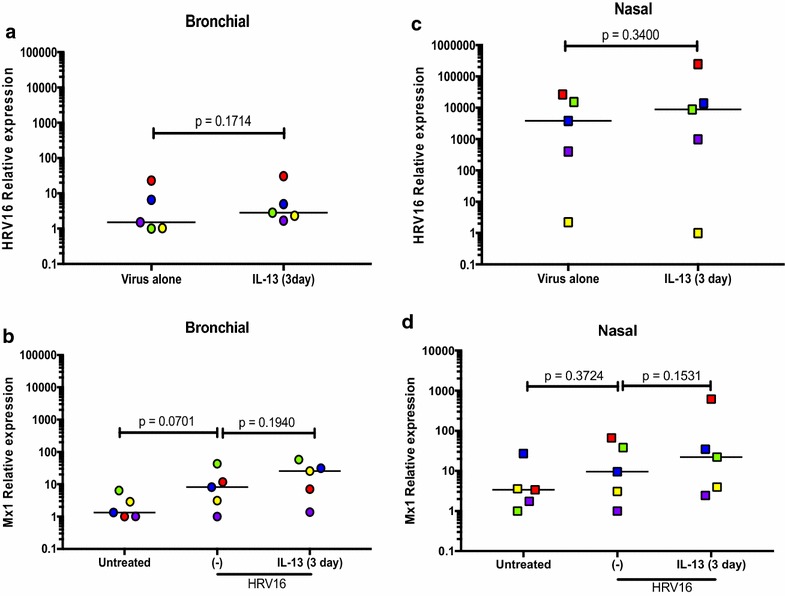
Effects of IL-13 treatment on human rhinovirus 16 (HRV16) infection in paired human primary bronchial and nasal epithelial cells under the submerged culture. Viral load and Mx1 mRNA expression were measured in bronchial (**a**, **b**) and nasal (**c**, **d**) epithelial cells exposed to IL-13 and/or HRV16. Each of the colored solid circles (n = 5 subjects) represents the data from an individual subject. The horizontal lines indicate medians

In the submerged culture, bronchial epithelial cells did not increase IP-10 production following HRV16 infection in the absence or presence of IL-13 treatment (Fig. 5a), although IL-13 treatment trended to increase IP-10 in the infected cells (p = 0.16). Unlike the ALI culture data, IL-13 treatment did not significantly change the viral load or Mx1 expression (Fig. 6a, b).

### Effects of IL-13 treatment on IP-10 expression and viral load in nasal epithelial cells

In the ALI culture, viral infection increased IP-10 production (Fig. 3b). Like the bronchial epithelial cells, IL-13 alone in the ALI culture did not significantly affect IP-10 production in nasal epithelial cells. Neither acute nor chronic IL-13 treatment changed IP-10 in the presence of infection. Viral load was affected by chronic IL-13 treatment in that it decreased similarly to the bronchial cells compared to the infected control (Fig. 4c). Like the bronchial cells, acute IL-13 treatment did not have an effect on viral load in nasal epithelial cells. (Fig. 4d).

In the submerged culture, RV16-infected cells from 4 out of the 5 subjects increased IP-10 production, while one subject’s cells decreased IP-10 (Fig. 5b). IL-13 treatment trended to increase viral load and Mx1 data showed a similar trend (Fig. 6c, d).

### Comparison of viral load and antiviral gene expression in nasal and bronchial epithelial cells

In the ALI culture, acute IL-13 treatment had minimal effect on viral load and Mx1 mRNA expression in both bronchial and nasal epithelial cells (Fig. 4). However, the chronic IL-13 treatment of both types of epithelial cells reduced viral load, particularly in nasal epithelial cells. Mx1 mRNA expression followed the trend of HRV16 changes in bronchial cells, but not in nasal cells.

In the submerged culture, IL-13 trended to increase HRV load in both bronchial and nasal epithelial cells (Fig. 6). Similarly, IL-13 increased Mx1 mRNA expression in both types of cells.

### Eotaxin 3 responses in IL-13 treated nasal and bronchial epithelial cells

**Fig. 7:**
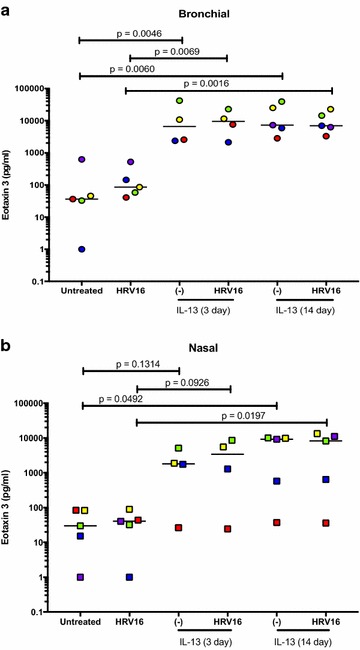
Effects of IL-13 treatment and human rhinovirus 16 (HRV16) infection on eotaxin 3 production in air–liquid interface (ALI) cultures of paired human primary bronchial (**a**) and nasal (**b**) epithelial cells. Each of the colored solid circles (n = 4 to 5 subjects) represents the data from an individual subject. The horizontal lines indicate medians

In the ALI culture of bronchial epithelial cells, IL-13 (acute and chronic) alone, but not HRV16 alone, significantly increased eotaxin 3 production (Fig. 7a). Nasal epithelial cells in the presence of IL-13 (acute and chronic) also increased eotaxin 3 (Fig. 7b). HRV16 infection in IL-13-treated cells of both cell types did not further increase eotaxin 3 production. Moreover, eotaxin 3 levels were similar between cells with acute and chronic IL-13 treatment.

**Fig. 8:**
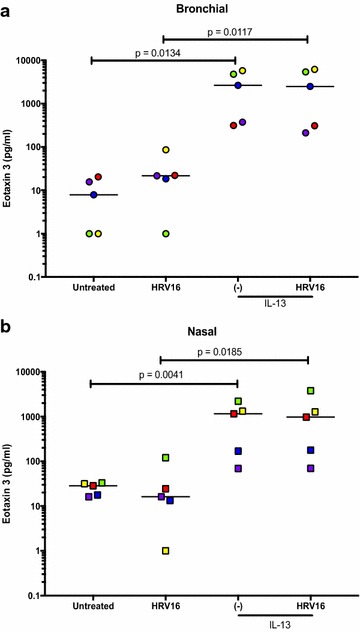
Effects of IL-13 treatment and human rhinovirus 16 (HRV16) infection on eotaxin 3 production paired human primary bronchial (**a**) and nasal (**b**) epithelial cells under the submerged culture. Each of the colored solid circles (n = 5 subjects) represents the data from an individual subject. The horizontal lines indicate medians

In the submerged culture of IL-13 treatment and viral infection, eotaxin 3 responses (Fig. 8a, b) in both nasal and bronchial epithelial cells followed the same pattern as described above for the ALI culture.

## Discussion

By using paired nasal and bronchial epithelial cells from subjects with various health conditions, we compared their responses to rhinovirus infection in the absence and presence of IL-13 treatment. Inclusion of human subjects with various disease states in the current study was aimed to broaden the implications of our research findings regarding the use of nasal epithelial cells in the research of various lung diseases. We found that both cell types have similar antiviral and pro-inflammatory responses. Specifically, nasal epithelial cells demonstrated similar IP-10, Mx1 and eotaxin 3 responses as bronchial cells to rhinovirus and/or IL-13 under ALI culture or submerged culture conditions.

Whether nasal epithelial cells can be used as a minimally invasive alternative to bronchial epithelial cells in the study of pathogenesis of asthma or other lung diseases remains uncertain. In our current study, we attempted to clarify this question by providing the following data. First, we utilized the cell culture model combining rhinovirus infection and IL-13 treatment, and found that viral infection in both cell types increased the expression of IP-10, although the increase was only significant in the ALI culture [15]. In the literature, both submerged and ALI cultures have been used to study the mechanisms of rhinovirus infection. Their results are not always consistent. To clarify if the discrepancies of research data are related to the culture methods, it is important to perform both cultures in the same subjects. Second, the effect of IL-13 on viral load is similar in both cell types. Third, eotaxin 3 production following IL-13 treatment in both cell types is similar in the absence or presence of viral infection. Collectively, our data suggest that in the setting of airway epithelial exposure to rhinovirus 16 and type 2 cytokine IL-13, nasal epithelial cells may serve as an alternative to the invasive bronchial brushing procedure. However, future studies are necessary to further validate the use of nasal cells in studying other pathogenic conditions of lower airways such as exposure to additional cytokines, allergens and other pathogens including respiratory viruses.

Our current study is unique in several aspects. First, we performed both ALI and submerged cultures in order to clarify the controversial data in the literature regarding the use of nasal epithelial cells in the study of lower airway diseases. Comer et al. [16] used both submerged and ALI cultures to test the response of bronchial and nasal cells from COPD patients to LPS and cigarette smoke extract, and concluded that nasal cells could not be surrogates for bronchial cells based on their different levels of production of pro-inflammatory cytokines. However, our data support the use of nasal cells to indicate the responses of bronchial epithelial cells to rhinovirus infection. Thus, the types of treatments may affect the conclusions. Second, we sought to address the controversy about the effects of IL-13 on viral load and the antiviral genes such as Mx1. Some studies have found IL-13 increases viral load in airway epithelial cells in ALI cultures [3]. Our findings in ALI cultures are contradictory to this publication in that a decrease in viral load was observed in both nasal and bronchial epithelial cells exposed to chronic, but not acute IL-13 treatments. However, in submerged culture, we found the opposite results because treatment with IL-13 trended to increase viral load. The decrease in viral load in ALI cultures could be due to the increased amount of mucous goblet cells in chronic IL-13-treated cells [17, 18]. These findings may not support the idea that asthmatic patients are more susceptible to viral infection because of a decreased interferon response [19, 20]. However, our data are consistent with those showing similar levels of viruses in airways of HRV-infected asthmatics with an exacerbation as compared to the normal subjects with HRV infection [21]. Murine studies also showed similar effects of IL-13 on viral titer of other strains of respiratory viruses. Zhou W et al. [22] demonstrated that IL-13 transgenic (overexpressing) mice decreases respiratory syncytial virus (RSV) titer along with IFN-γ production in the lungs compared to the wild-type mice. Likewise, IL-13 knockout mice showed an increase in RSV titer.

There were heterogeneous responses of two cell types to HRV, even from the same subject. The heterogeneous responses were seen in both viral load and Mx1 expression. We speculate that these may be related to the degree of their susceptibility to HRV infection. Previous studies suggest that rhinoviruses replicate favorably at a lower temperature in the nasal cavity. Mechanistically, a study of mouse airway epithelial cell culture with HRV1B infection demonstrated that low temperature (i.e., 33°C) vs. warm temperature (i.e., 37 °C) allows more viral replication due to less induction of antiviral genes [23]. In our current study, we initially infected both nasal and bronchial epithelial cells cultured at the ALI for 90 min at 34.5 °C, a temperature reflecting that in the nasal cavity. We then maintained the cell culture at 37 °C, a temperature best supporting the growth of epithelial cells. Temperature and differing antiviral gene levels could have resulted in slightly different responses from each cell type in some subjects. Variation in baseline Mx1 expression, as reported in the literature [24], between the two cell types could be another contributing factor in heterogeneous responses.

We determined the effect of IL-13 on the production of IP-10 which participates in the recruitment of inflammatory cells such as T cells and macrophages [7, 25]. As IP-10 is considered a biomarker for active rhinovirus infection in asthma [7, 25], it is important to uncover if IL-13 has any impact on IP-10 production in both injured (basal cells maintained in submerged cell culture) [13, 14] and intact (ALI cultured well-differentiated cells) airway epithelial cells. Although HRV16 significantly induced IP-10 production in ALI cultures, acute or chronic IL-13 treatment had no significant effect on IP-10 levels in both nasal and bronchial epithelial cells under submerged and ALI conditions. This finding is intriguing given the fact that rhinovirus alone increased IP-10, and IL-13 chronic treatment in virus-infected ALI cultured cells decreases viral load. We speculate that viral load is mainly controlled by the amount of goblet cells induced by IL-13, while IP-10 production may come from non-goblet cells (e.g., ciliated cells) that are more prone to viral infection with subsequent IP-10 production. Such a hypothesis could be tested in future studies using other experimental approaches such as single cell RNA sequencing to define the co-expression of the viral gene and IP-10 or other antiviral genes in different subtypes of nasal and bronchial epithelial cells under ALI cultures.

In addition, we determined if rhinoviruses had an impact on IL-13-induced eotaxin 3 production in paired nasal and bronchial epithelial cells. As eotaxin 3 is critical to airway eosinophilic inflammation [26], our data may unravel the role of viral infection in modulating eosinophilic inflammation. Although IL-13 clearly increased eotaxin 3 production, rhinoviruses had no significant effect on its levels in both nasal and bronchial epithelial cells, suggesting that viral infection may not further enhance eosinophilic inflammation in type 2 inflammation-high airways. We chose to measure eotaxin 3 in the current study because both eotaxin 2 and eotaxin 3 are similarly up-regulated by IL-13, and eotaxin 1 levels have not been shown to differ in normal subjects and asthmatics with the differing severity of the disease [27, 28]. Our finding of no up-regulation of eotaxin 3 by HRV infection is consistent with our recent publication [29]. Although HRV infection alone has been reported to increase eotaxin and eotaxin 2 [30], this finding is still controversial, as cell culture studies by other research groups have not been able to show the similar data [31, 32].

Our study used the rhinovirus 16 and IL-13 treatment model to test whether nasal epithelial cells could be used as surrogates for bronchial epithelial cells in order to reduce the risks of bronchoscopy and the burden on patients. We are aware that other experimental models such as those related to airway remodeling could be tested in the future to compare these two cell types. Also, this study does not address the mechanisms by which nasal and bronchial epithelial cells have similar responses to viral infection and IL-13 treatment. For example, it is unclear whether there is similar cellular composition between the cell types that could affect their responses to HRV16 and IL-13. Finally, whether findings from our cell culture studies can be applied to the more complex in vivo models warrants further investigation.

## Conclusions

In summary, our study shows the potential for nasal and bronchial airway epithelial cells to be used interchangeably based on the rhinovirus infection and IL-13 treatment model. Our findings would further justify the use of the minimally invasive nasal brushing approach in the basic and translational research of lower airways diseases.

## Abbreviations

ALI: air liquid interface
HRV16: human rhinovirus 16
IL-13: interleukin 13
PFU: plaque forming unit
IP-10: interferon gamma-induced protein 10
